# Crystal structure of methyl 1-allyl-4-methyl-1*H*-benzo[*c*][1,2]thia­zine-3-carboxyl­ate 2,2-dioxide

**DOI:** 10.1107/S2056989016015978

**Published:** 2016-10-14

**Authors:** Liliana Azotla-Cruz, Svitlana Shishkina, Igor Ukrainets, Irina Lijanova, Natalya Likhanova

**Affiliations:** a National Polytechnic Institute, CIITEC, Cerrada Cecati S/N, Colonia Santa Catarina de Azcapotzalco, CP 02250, Mexico, DF, Mexico; b SSI ‘Institute for Single Crystals’, National Academy of Sciences of Ukraine, 60 Lenina Avenue, Kharkiv 61001, Ukraine; c National University of Pharmacy, 4 Blyukhera St., Kharkiv 61168, Ukraine; d Mexican Institute of Petroleum, Eje Central Lazaro Cardenas Norte 152, Col. San Bartolo Atepehuacan, 07730 Mexico, DF, Mexico

**Keywords:** crystal structure, benzo­thia­zine, all­yl, C—H⋯π inter­actions

## Abstract

In the title compound, C_14_H_15_NO_4_S, the di­hydro­thia­zine ring adopts a distorted sofa conformation with the S atom displaced from the mean plane through the N and C ring atoms by 0.767 (1) Å. The allyl substituent (C—C=C) is inclined to this mean plane by 78.5 (7)° and the acetate group [C(=O)—O—C] by 66.5 (3)°. In the crystal, mol­ecules are linked by C—H⋯π inter­actions forming chains propagating along the *a*-axis direction.

## Abstract 2

In the title benzo­thia­zine compound, the di­hydro­thia­zine ring adopts a sofa-like conformation with the S atom displaced from the mean plane through the N and C ring atoms by 0.767 (1) Å. In the crystal, mol­ecules are linked by C—H⋯π inter­actions, forming chains propagating along the *a*-axis direction.

## Chemical context   

**Figure 1:**
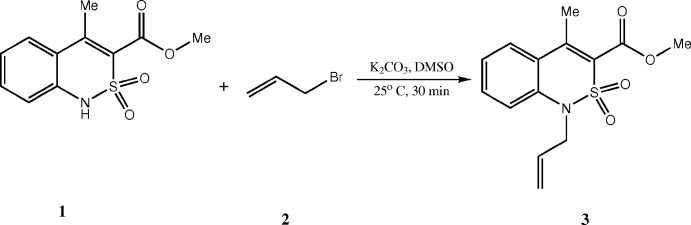
The synthesis of the title compound, **3**.

Alkyl­ation of nitro­gen heterocycles, particularly those containing reactive exocyclic groups, always attracts attention with its ambiguity and dependence on a variety of factors. For example, esters of 4-hy­droxy-2-oxo-1,2-di­hydro­quinoline-3-carb­oxy­lic acids are primarily alkyl­ated exclusively at the 4-OH group (Ukrainets *et al.*, 1996 ▸). However, methyl 4-hy­droxy-2,2-dioxo-1*H*-2λ^6^,1-benzo­thia­zine-3-carboxyl­ates that are structurally close to them easily form mixtures of isomeric 3-*C*- and 4-*O*-alkyl­ation products under the same conditions (Ukrainets *et al.*, 2015 ▸). Consequently, it is quite difficult to predict their behaviour in the alkyl­ation reactions of the esters of 4-methyl-2,2-dioxo-1*H*-2λ^6^,1-benzo­thia­zine-3-carb­oxy­lic acids, and the determination of the true structure is essential. It has been found that methyl 4-methyl-2,2-dioxo-1*H*-2λ^6^,1-benzo­thia­zine-3-carboxyl­ate **1** in the K_2_CO_3_/DMSO system is rapidly alkyl­ated with allyl bromide **2** by the cyclic nitro­gen atom, with formation of the main product of the reaction studied *viz.* compound **3** (see Fig. 1 ▸).

## Structural commentary   

**Figure 2:**
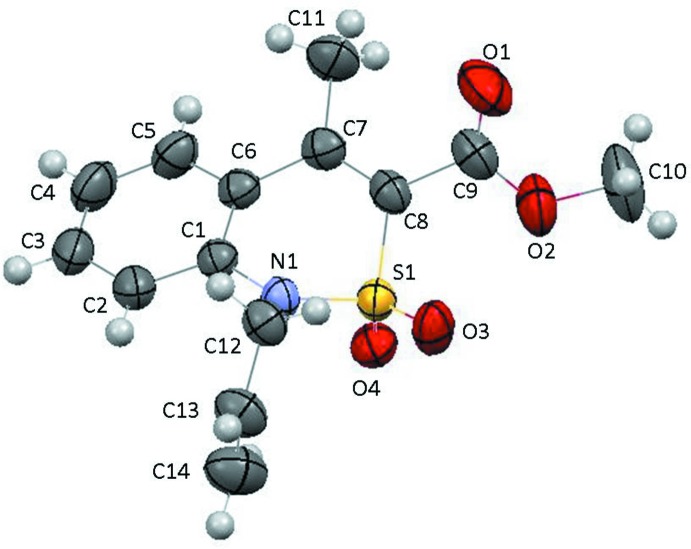
The mol­ecular structure of compound **3**, with atom labelling. Displacement ellipsoids are drawn at the 50% probability level.

The mol­ecular structure of the title compound, **3**, is illustrated in Fig. 2 ▸. The di­hydro­thia­zine ring adopts a distorted sofa conformation: the puckering parameters (Zefirov *et al.*, 1990 ▸) are: *S* = 0.67, Θ = 57.1°, Ψ = 19.0°. Atom S1 deviates from the mean plane of the remaining atoms (N1/C1/C6–C8) by 0.767 (1) Å. The allyl substituent (C—C=C) is inclined to this mean plane by 78.5 (7)° and the acetate group (O=C—O—C) by 66.5 (3)°. Atom N1 has a planar configuration, the sum of the bond angles being 359.1°.
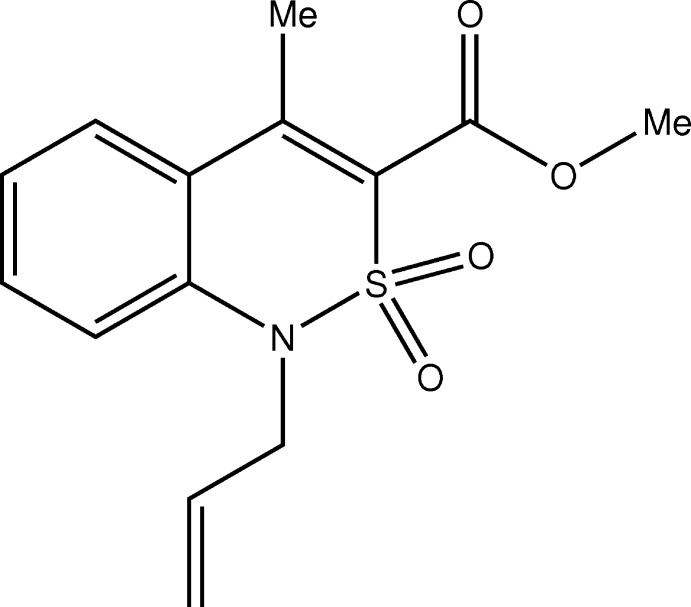


The strong steric repulsion between methyl group at the C7 atom and the aromatic ring {there are short intra­molecular contacts H5⋯C11 = 2.63 and H11*A*⋯C5 = 2.47 Å in this fragment [the sum of the van der Waals radii (Zefirov, 1997 ▸) is 2.87 Å]} causes a disturbance of the conjugation between the π-systems of the aromatic ring and the C7=C8 double bond; the C5—C6—C7—C8 torsion angle is −164.7 (4)°. The ester substituent is twisted relatively to the C7=C8 endocyclic double bond [the C7—C8—C9—O1 torsion angle is 46.0 (7)°], leading to its elongation: the C7=C8 bond length is 1.348 (6) Å as compared to the mean value of 1.326 Å (Bürgi & Dunitz, 1994 ▸). The methyl group of the ester substituent is located in the *ap*-position to the C8—C9 bond [C8—C9—O2—C10 = −171.5 (5)°]. The allyl group is orthogonal to the benzo­thia­zine fragment plane while the terminal double bond is synperiplanar to the N1—C12 bond [torsion angles C1—N1—C12—C13 and N1—C12—C13—C14 are 97.2 (5) and 3.5 (8)°, respectively]. The steric repulsion between the allyl substituent and the aromatic cycle (short intra­molecular contacts H2⋯C12 = 2.77 Å and H12*A*⋯C2 = 2.83 Å) results in the elongation of the C1—N1 bond [1.411 (5) Å], compared with the mean value of 1.371 Å (Bürgi & Dunitz, 1994 ▸).

## Supra­molecular features   

**Figure 3:**
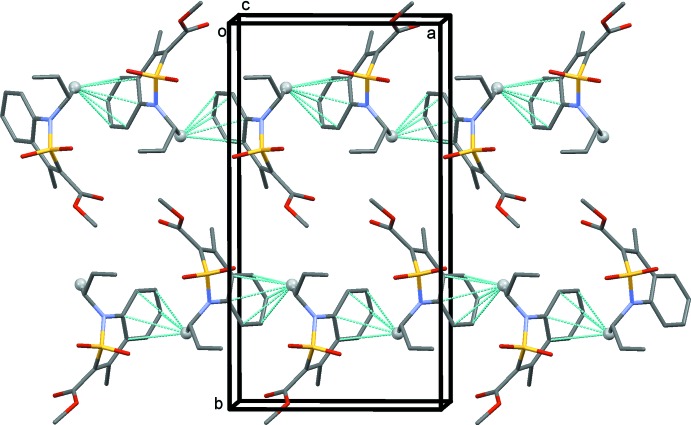
A view along the *c* axis of the crystal packing of compound **3**. The C—H⋯π inter­actions are represented by dashed lines (see Table 1 ▸) and, for clarity, only H atom H12*A* (grey ball) is included.

In the crystal, mol­ecules are linked by C—H⋯π inter­actions, forming chains propagating along the *a*-axis direction. (Table 1 ▸ and Fig. 3 ▸). There are no other significant inter­molecular inter­actions in the crystal structure, despite the presence of a number of potential donor and acceptor atoms.

## Database survey   

**Figure 4:**
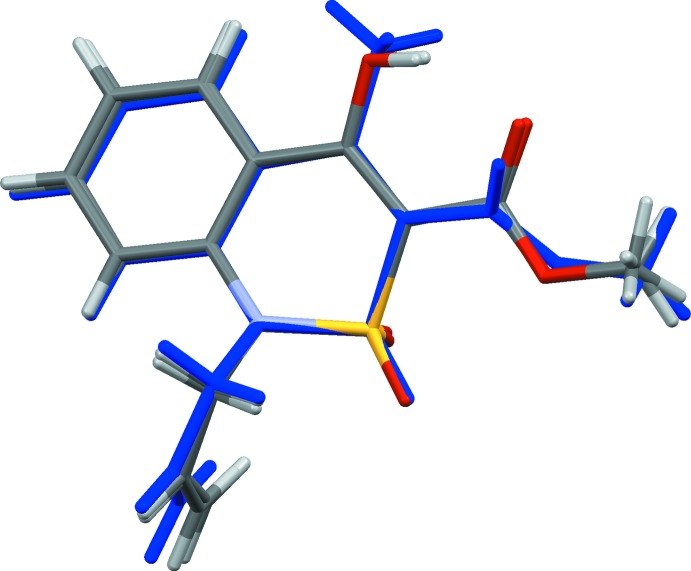
The structural overlap of the two independent mol­ecules of the 4-hy­droxy analogue (MINJAW; Shishkina *et al.*, 2013 ▸), and the title compound **3**, shown in blue.

A search of the Cambridge Structural Database (Version 5.37, update May 2016; Groom *et al.*, 2016 ▸) for 1*H*-benzo[*c*][1,2]thia­zine 2,2-dioxide yielded 15 hits. These include the 4-hy­droxy analogue of the title compound, *viz.* methyl 1-allyl-4-hy­droxy-1*H*-benzo[*c*][1,2]thia­zine-3-carboxyl­ate 2,2-dioxide (MINJAW; Shishkina *et al.*, 2013 ▸). This compound crystallized with two mol­ecules in the asymmetric unit. The conformation of the di­hydro­thia­zine ring in both mol­ecules resembles that in the title compound, which has a distorted sofa conformation. A view of the structural overlap of the three mol­ecules is shown in Fig. 4 ▸.

## Synthesis and crystallization   

The synthesis of the title compound, **3**, is illustrated in Fig. 1 ▸. To a solution of 2.53 g (0.01 mol) of methyl 4-methyl-2,2-dioxo-1*H*-2λ^6^,1-benzo­thia­zine-3-carboxyl­ate, **1**, in 20 ml DMSO were added 2.07 g (0.015 mol) of K_2_CO_3_ and the mixture was stirred for 30 min. Allyl bromide (1.81 g, 0.015 mol) was then added and the mixture was stirred for a further 30 min at 298 K. It was then diluted with cold water and acidified with dilute HCl to pH 4. It was extracted with CH_2_Cl_2_ (3 × 10 ml). The organic extracts were combined and the solvent removed by distillation (at reduced pressure at the end). The residue was dissolved in 20 ml of hot methanol and filtered over charcoal. The resulting solution was then placed in a freezer (253 K) for 24 h, after which crystals of the title compound were harvested (yield 2.55 g, 87%; m.p. 360–362 K).

## Refinement   

Crystal data, data collection and structure refinement details are summarized in Table 2 ▸. H atoms could all be located in difference Fourier maps. During refinement they were included in calculated positions and treated as riding: C—H = 0.93–0.97 Å with *U*_iso_ = 1.5*U*_eq_(C-meth­yl) and 1.2*U*_eq_(C) for other H atoms.

## Supplementary Material

Crystal structure: contains datablock(s) I, Global. DOI: 10.1107/S2056989016015978/su5329sup1.cif

Structure factors: contains datablock(s) I. DOI: 10.1107/S2056989016015978/su5329Isup2.hkl

Supporting information file. DOI: 10.1107/S2056989016015978/su5329Isup3.cml

CCDC reference: 1508990

Additional supporting information: crystallographic information; 3D view; checkCIF report

## Figures and Tables

**Table 1:** Hydrogen-bond geometry (Å, °) *Cg* is the centroid of the C1–C6 ring.

*D*—H⋯*A*	*D*—H	H⋯*A*	*D*⋯*A*	*D*—H⋯*A*
C12—H12*A*⋯*Cg* ^i^	0.97	2.95	3.576 (5)	123

Symmetry code: (i) 
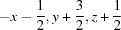
.

**Table 2:** Experimental details

Crystal data	
Chemical formula	C_14_H_15_NO_4_S
*M*_r_	293.33
Crystal system, space group	Orthorhombic, *P* *n* *a*2_1_
Temperature (K)	293
*a*, *b*, *c* (Å)	10.1970 (7), 18.6174 (12), 7.5136 (5)
*V* (Å^3^)	1426.39 (16)
*Z*	4
Radiation type	Mo *K*α
μ (mm^−1^)	0.24
Crystal size (mm)	0.20 × 0.10 × 0.02

Data collection	
Diffractometer	Agilent Xcalibur Sapphire3
Absorption correction	Multi-scan (*CrysAlis RED*; Agilent, 2012 ▸)
*T*_min_, *T*_max_	0.706, 1.000
No. of measured, independent and observed [*I* > 2σ(*I*)] reflections	9346, 2425, 2111
*R*_int_	0.056
(sin θ/λ)_max_ (Å^−1^)	0.595

Refinement	
*R*[*F* ^2^ > 2σ(*F* ^2^)], *wR*(*F* ^2^), *S*	0.044, 0.120, 1.05
No. of reflections	2425
No. of parameters	181
No. of restraints	1
H-atom treatment	H-atom parameters constrained
Δρ_max_, Δρ_min_ (e Å^−3^)	0.19, −0.20
Absolute structure	Flack *x* determined using 785 quotients [(*I* ^+^)−(*I* ^−^)]/[(*I* ^+^)+(*I* ^−^)] (Parsons *et al.*, 2013 ▸)
Absolute structure parameter	0.09 (8)

Computer programs: *CrysAlis CCD* and *CrysAlis RED* (Agilent, 2012 ▸), *SHELXS2014* (Sheldrick, 2008 ▸), *SHELXTL* (Sheldrick, 2008 ▸), *Mercury* (Macrae *et al.*, 2008 ▸), *SHELXL2014* (Sheldrick, 2015 ▸), *PLATON* (Spek, 2009 ▸) and *publCIF* (Westrip, 2010 ▸).

## supplementary crystallographic information

### Crystal data

**Table:**

C_14_H_15_NO_4_S	*D*_x_ = 1.366 Mg m^−^^3^
*M**_r_* = 293.33	Mo *K*α radiation, λ = 0.71073 Å
Orthorhombic, *P**n**a*2_1_	Cell parameters from 2163 reflections
*a* = 10.1970 (7) Å	θ = 3.8–25.1°
*b* = 18.6174 (12) Å	µ = 0.24 mm^−^^1^
*c* = 7.5136 (5) Å	*T* = 293 K
*V* = 1426.39 (16) Å^3^	Plate, colourless
*Z* = 4	0.20 × 0.10 × 0.02 mm
*F*(000) = 616	

### Data collection

**Table:**

Agilent Xcalibur Sapphire3 diffractometer	2425 independent reflections
Radiation source: Enhance (Mo) X-ray Source	2111 reflections with *I* > 2σ(*I*)
Detector resolution: 16.1827 pixels mm^-1^	*R*_int_ = 0.056
ω–scans	θ_max_ = 25.0°, θ_min_ = 3.0°
Absorption correction: multi-scan (CrysAlis RED; Agilent, 2012)	*h* = −12→10
*T*_min_ = 0.706, *T*_max_ = 1.000	*k* = −22→22
9346 measured reflections	*l* = −7→8

### Refinement

**Table:**

Refinement on *F*^2^	Secondary atom site location: difference Fourier map
Least-squares matrix: full	Hydrogen site location: inferred from neighbouring sites
*R*[*F*^2^ > 2σ(*F*^2^)] = 0.044	H-atom parameters constrained
*wR*(*F*^2^) = 0.120	*w* = 1/[σ^2^(*F*_o_^2^) + (0.0618*P*)^2^ + 0.1291*P*] where *P* = (*F*_o_^2^ + 2*F*_c_^2^)/3
*S* = 1.05	(Δ/σ)_max_ < 0.001
2425 reflections	Δρ_max_ = 0.19 e Å^−^^3^
181 parameters	Δρ_min_ = −0.20 e Å^−^^3^
1 restraint	Absolute structure: Flack *x* determined using 785 quotients [(*I*^+^)-(*I*^-^)]/[(*I*^+^)+(*I*^-^)] (Parsons *et al.*, 2013)
Primary atom site location: structure-invariant direct methods	Absolute structure parameter: 0.09 (8)

### Special details

**Table:**

Geometry. All esds (except the esd in the dihedral angle between two l.s. planes) are estimated using the full covariance matrix. The cell esds are taken into account individually in the estimation of esds in distances, angles and torsion angles; correlations between esds in cell parameters are only used when they are defined by crystal symmetry. An approximate (isotropic) treatment of cell esds is used for estimating esds involving l.s. planes.

### Fractional atomic coordinates and isotropic or equivalent isotropic displacement parameters (Å^2^)

**Table:**

	*x*	*y*	*z*	*U*_iso_*/*U*_eq_	
S1	0.88243 (9)	0.65549 (5)	0.26457 (17)	0.0457 (3)	
O1	0.6502 (4)	0.5240 (2)	0.4865 (7)	0.0975 (14)	
O2	0.7649 (4)	0.51032 (18)	0.2347 (6)	0.0801 (11)	
O3	0.7789 (3)	0.66144 (16)	0.1376 (5)	0.0645 (9)	
O4	1.0109 (3)	0.63899 (17)	0.2033 (4)	0.0590 (9)	
N1	0.8881 (3)	0.73110 (17)	0.3775 (5)	0.0457 (8)	
C1	0.9692 (4)	0.7307 (2)	0.5295 (6)	0.0443 (9)	
C2	1.0333 (4)	0.7933 (3)	0.5818 (7)	0.0559 (11)	
H2	1.0205	0.8355	0.5180	0.067*	
C3	1.1146 (4)	0.7932 (3)	0.7260 (7)	0.0652 (14)	
H3	1.1557	0.8355	0.7611	0.078*	
C4	1.1362 (5)	0.7308 (3)	0.8195 (7)	0.0722 (15)	
H4	1.1956	0.7305	0.9135	0.087*	
C5	1.0705 (5)	0.6690 (3)	0.7747 (8)	0.0605 (11)	
H5	1.0837	0.6277	0.8419	0.073*	
C6	0.9842 (4)	0.6670 (2)	0.6302 (6)	0.0461 (10)	
C7	0.9017 (4)	0.6034 (2)	0.5943 (6)	0.0490 (10)	
C8	0.8424 (4)	0.5951 (2)	0.4353 (6)	0.0497 (11)	
C9	0.7425 (5)	0.5388 (2)	0.3921 (8)	0.0623 (13)	
C10	0.6651 (7)	0.4617 (4)	0.1624 (12)	0.110 (3)	
H10A	0.6927	0.4446	0.0478	0.165*	
H10B	0.6537	0.4217	0.2415	0.165*	
H10C	0.5835	0.4870	0.1506	0.165*	
C11	0.8826 (5)	0.5494 (3)	0.7415 (9)	0.0715 (15)	
H11A	0.9319	0.5640	0.8442	0.107*	
H11B	0.7913	0.5467	0.7717	0.107*	
H11C	0.9126	0.5031	0.7025	0.107*	
C12	0.7970 (4)	0.7897 (2)	0.3411 (7)	0.0547 (11)	
H12A	0.7753	0.8127	0.4530	0.066*	
H12B	0.7168	0.7693	0.2936	0.066*	
C13	0.8440 (5)	0.8458 (2)	0.2154 (8)	0.0661 (15)	
H13	0.7888	0.8848	0.1961	0.079*	
C14	0.9528 (6)	0.8455 (3)	0.1316 (9)	0.0829 (18)	
H14A	1.0112	0.8076	0.1466	0.099*	
H14B	0.9737	0.8832	0.0555	0.099*	

### Atomic displacement parameters (Å^2^)

**Table:**

	*U*^11^	*U*^22^	*U*^33^	*U*^12^	*U*^13^	*U*^23^
S1	0.0486 (5)	0.0440 (5)	0.0446 (5)	−0.0024 (4)	−0.0040 (5)	−0.0029 (5)
O1	0.087 (3)	0.089 (3)	0.116 (4)	−0.038 (2)	0.020 (3)	−0.007 (3)
O2	0.084 (2)	0.063 (2)	0.094 (3)	−0.0162 (18)	−0.005 (2)	−0.028 (2)
O3	0.070 (2)	0.062 (2)	0.062 (2)	−0.0037 (16)	−0.0255 (17)	−0.0033 (16)
O4	0.0587 (18)	0.0636 (19)	0.0546 (19)	−0.0032 (15)	0.0108 (14)	−0.0081 (14)
N1	0.0528 (19)	0.0364 (17)	0.048 (2)	−0.0005 (14)	−0.0052 (15)	−0.0030 (17)
C1	0.044 (2)	0.046 (2)	0.043 (2)	−0.0011 (18)	0.0033 (18)	−0.0031 (18)
C2	0.061 (3)	0.053 (3)	0.053 (3)	−0.008 (2)	0.004 (2)	−0.005 (2)
C3	0.070 (3)	0.075 (3)	0.051 (3)	−0.020 (3)	0.005 (2)	−0.015 (2)
C4	0.075 (3)	0.096 (4)	0.046 (3)	−0.009 (3)	−0.013 (2)	−0.008 (3)
C5	0.070 (3)	0.067 (3)	0.045 (2)	0.005 (2)	−0.009 (3)	0.001 (3)
C6	0.049 (2)	0.047 (2)	0.042 (2)	0.0024 (18)	0.0034 (18)	−0.0002 (18)
C7	0.055 (2)	0.042 (2)	0.050 (3)	0.0070 (18)	0.003 (2)	0.0025 (19)
C8	0.051 (2)	0.038 (2)	0.060 (3)	−0.0031 (18)	0.003 (2)	−0.0031 (19)
C9	0.062 (3)	0.046 (2)	0.079 (4)	−0.009 (2)	0.000 (3)	0.000 (2)
C10	0.112 (5)	0.070 (4)	0.149 (7)	−0.028 (4)	−0.040 (5)	−0.028 (4)
C11	0.093 (4)	0.053 (3)	0.068 (4)	−0.001 (2)	−0.002 (3)	0.014 (3)
C12	0.054 (2)	0.046 (2)	0.064 (3)	0.0025 (19)	−0.001 (2)	0.001 (2)
C13	0.067 (3)	0.051 (3)	0.081 (4)	0.003 (2)	0.000 (3)	0.011 (2)
C14	0.076 (4)	0.090 (4)	0.082 (4)	−0.001 (3)	−0.001 (3)	0.026 (3)

### Geometric parameters (Å, º)

**Table:**

S1—O4	1.422 (3)	C5—H5	0.9300
S1—O3	1.427 (3)	C6—C7	1.477 (6)
S1—N1	1.645 (3)	C7—C8	1.348 (6)
S1—C8	1.754 (4)	C7—C11	1.508 (7)
O1—C9	1.210 (6)	C8—C9	1.497 (6)
O2—C9	1.316 (6)	C10—H10A	0.9600
O2—C10	1.466 (6)	C10—H10B	0.9600
N1—C1	1.411 (5)	C10—H10C	0.9600
N1—C12	1.458 (5)	C11—H11A	0.9600
C1—C2	1.392 (6)	C11—H11B	0.9600
C1—C6	1.415 (6)	C11—H11C	0.9600
C2—C3	1.364 (7)	C12—C13	1.487 (7)
C2—H2	0.9300	C12—H12A	0.9700
C3—C4	1.376 (8)	C12—H12B	0.9700
C3—H3	0.9300	C13—C14	1.276 (8)
C4—C5	1.373 (7)	C13—H13	0.9300
C4—H4	0.9300	C14—H14A	0.9300
C5—C6	1.398 (6)	C14—H14B	0.9300
			
O4—S1—O3	118.8 (2)	C7—C8—C9	125.3 (4)
O4—S1—N1	108.66 (18)	C7—C8—S1	118.0 (3)
O3—S1—N1	107.72 (19)	C9—C8—S1	116.6 (4)
O4—S1—C8	108.2 (2)	O1—C9—O2	124.8 (5)
O3—S1—C8	111.5 (2)	O1—C9—C8	124.1 (5)
N1—S1—C8	100.3 (2)	O2—C9—C8	111.0 (4)
C9—O2—C10	117.4 (5)	O2—C10—H10A	109.5
C1—N1—C12	122.0 (3)	O2—C10—H10B	109.5
C1—N1—S1	115.7 (3)	H10A—C10—H10B	109.5
C12—N1—S1	121.4 (3)	O2—C10—H10C	109.5
C2—C1—N1	120.0 (4)	H10A—C10—H10C	109.5
C2—C1—C6	120.0 (4)	H10B—C10—H10C	109.5
N1—C1—C6	120.0 (3)	C7—C11—H11A	109.5
C3—C2—C1	120.5 (5)	C7—C11—H11B	109.5
C3—C2—H2	119.7	H11A—C11—H11B	109.5
C1—C2—H2	119.7	C7—C11—H11C	109.5
C2—C3—C4	120.3 (5)	H11A—C11—H11C	109.5
C2—C3—H3	119.9	H11B—C11—H11C	109.5
C4—C3—H3	119.9	N1—C12—C13	116.1 (4)
C5—C4—C3	120.3 (5)	N1—C12—H12A	108.3
C5—C4—H4	119.9	C13—C12—H12A	108.3
C3—C4—H4	119.9	N1—C12—H12B	108.3
C4—C5—C6	121.4 (5)	C13—C12—H12B	108.3
C4—C5—H5	119.3	H12A—C12—H12B	107.4
C6—C5—H5	119.3	C14—C13—C12	126.2 (5)
C5—C6—C1	117.4 (4)	C14—C13—H13	116.9
C5—C6—C7	121.5 (4)	C12—C13—H13	116.9
C1—C6—C7	120.8 (4)	C13—C14—H14A	120.0
C8—C7—C6	120.7 (4)	C13—C14—H14B	120.0
C8—C7—C11	121.0 (4)	H14A—C14—H14B	120.0
C6—C7—C11	118.3 (4)		
			
O4—S1—N1—C1	−60.7 (3)	C1—C6—C7—C8	21.7 (6)
O3—S1—N1—C1	169.5 (3)	C5—C6—C7—C11	17.0 (6)
C8—S1—N1—C1	52.7 (3)	C1—C6—C7—C11	−156.7 (4)
O4—S1—N1—C12	130.1 (3)	C6—C7—C8—C9	−171.1 (4)
O3—S1—N1—C12	0.2 (4)	C11—C7—C8—C9	7.3 (7)
C8—S1—N1—C12	−116.5 (3)	C6—C7—C8—S1	7.8 (6)
C12—N1—C1—C2	−42.8 (5)	C11—C7—C8—S1	−173.9 (4)
S1—N1—C1—C2	147.9 (3)	O4—S1—C8—C7	72.8 (4)
C12—N1—C1—C6	136.3 (4)	O3—S1—C8—C7	−154.8 (3)
S1—N1—C1—C6	−32.9 (5)	N1—S1—C8—C7	−40.9 (4)
N1—C1—C2—C3	−178.4 (4)	O4—S1—C8—C9	−108.2 (4)
C6—C1—C2—C3	2.5 (6)	O3—S1—C8—C9	24.2 (4)
C1—C2—C3—C4	1.2 (7)	N1—S1—C8—C9	138.1 (3)
C2—C3—C4—C5	−3.6 (8)	C10—O2—C9—O1	5.4 (8)
C3—C4—C5—C6	2.4 (8)	C10—O2—C9—C8	−171.5 (5)
C4—C5—C6—C1	1.2 (7)	C7—C8—C9—O1	46.0 (7)
C4—C5—C6—C7	−172.6 (5)	S1—C8—C9—O1	−132.9 (5)
C2—C1—C6—C5	−3.6 (6)	C7—C8—C9—O2	−137.1 (5)
N1—C1—C6—C5	177.2 (4)	S1—C8—C9—O2	44.0 (5)
C2—C1—C6—C7	170.3 (4)	C1—N1—C12—C13	97.2 (5)
N1—C1—C6—C7	−8.9 (6)	S1—N1—C12—C13	−94.2 (4)
C5—C6—C7—C8	−164.7 (4)	N1—C12—C13—C14	3.5 (8)

### Hydrogen-bond geometry (Å, º)

Cg is the centroid of the C1–C6 ring.

**Table:**

*D*—H···*A*	*D*—H	H···*A*	*D*···*A*	*D*—H···*A*
C12—H12*A*···*Cg*^i^	0.97	2.95	3.576 (5)	123

Symmetry code: (i) −*x*−1/2, *y*+3/2, *z*+1/2.
